# Carbon monoxide-dependent transcriptional changes in a thermophilic, carbon monoxide-utilizing, hydrogen-evolving bacterium *Calderihabitans maritimus* KKC1 revealed by transcriptomic analysis

**DOI:** 10.1007/s00792-020-01175-z

**Published:** 2020-05-09

**Authors:** Masao Inoue, Hikaru Izumihara, Yuto Fukuyama, Kimiho Omae, Takashi Yoshida, Yoshihiko Sako

**Affiliations:** Graduate School of Agriculture, Kyoto University, Kitashirakawa Oiwake-cho, Sakyo-ku, Kyoto, 606-8502 Japan

**Keywords:** Carbon monoxide, Hydrogen, Hydrogenase, Energy conservation, Transcriptome, RNA-seq

## Abstract

*Calderihabitans maritimus* KKC1 is a thermophilic, carbon monoxide (CO)-utilizing, hydrogen-evolving bacterium that harbors seven *cooS* genes for anaerobic CO dehydrogenases and six *hyd* genes for [NiFe] hydrogenases and capable of using a variety of electron acceptors coupled to CO oxidation. To understand the relationships among these unique features and the transcriptional adaptation of the organism to CO, we performed a transcriptome analysis of *C. maritimus* KKC1 grown under 100% CO and N_2_ conditions. Of its 3114 genes, 58 and 32 genes were significantly upregulated and downregulated in the presence of CO, respectively. A *cooS–ech* gene cluster, an “orphan” *cooS* gene, and bidirectional *hyd* genes were upregulated under CO, whereas hydrogen-uptake *hyd* genes were downregulated. Transcriptional changes in anaerobic respiratory genes supported the broad usage of electron acceptors in *C. maritimus* KKC1 under CO metabolism. Overall, the majority of the differentially expressed genes were oxidoreductase-like genes, suggesting metabolic adaptation to the cellular redox change upon CO oxidation. Moreover, our results suggest a transcriptional response mechanism to CO that involves multiple transcription factors, as well as a CO-responsive transcriptional activator (CooA). Our findings shed light on the diverse mechanisms for transcriptional and metabolic adaptations to CO in CO-utilizing and hydrogen-evolving bacteria.

**Electronic supplementary material:**

The online version of this article (10.1007/s00792-020-01175-z) contains supplementary material, which is available to authorized users.

## Introduction

Carbon monoxide (CO) is used as an energy source by CO-oxidizing microbes (carboxydotrophs) because of its low redox potential (Ragsdale 2004; Oelgeschläger and Rother 2008; Sokolova et al. 2009; Diender et al. 2015). Carboxydotrophs harness CO dehydrogenases (CODHs) for CO utilization by catalyzing the reaction CO + H_2_O ⇌ CO_2_ + 2H^+^  + 2e^−^ (Ragsdale 2004; Oelgeschläger and Rother 2008). CODHs are divided into two families: anaerobic Ni-containing CODHs (Ni-CODHs) and aerobic molybdenum- and copper-containing CODHs (Can et al. 2014; Hille et al. 2015). Unlike aerobic CODHs, Ni-CODHs can reduce ferredoxin and thereby utilize various types of terminal electron acceptors, such as protons, CO_2_, sulfate, and ferric iron [Fe(III)] (Oelgeschläger and Rother 2008; Sokolova et al. 2009; Diender et al. 2015). Because of this unique feature of Ni-CODHs, physiologically diverse anaerobic carboxydotrophs have been described, such as hydrogenogens, acetogens, methanogens, sulfate reducers, and Fe(III) reducers (Oelgeschläger and Rother 2008; Sokolova et al. 2009; Diender et al. 2015).

Hydrogenogenic carboxydotrophs couple CO oxidation with proton reduction to produce hydrogen (H_2_), during which the proton- or sodium-motive force is generated with residual energy via a Ni-CODH/energy converting hydrogenase (ECH) complex (Singer et al. 2006; Schut et al. 2016; Schoelmerich and Müller 2019). CO-dependent H_2_ production by hydrogenogenic carboxydotrophs is considered a “safety valve” to reduce toxic CO and supply H_2_, which is an energy source for H_2_-utilizing microbial communities (Techtmann et al. 2009). Hydrogenogenic carboxydotrophs are generally divided into three groups in their phylogeny: Firmicutes, Proteobacteria, and Archaea (Diender et al. 2015; Inoue et al. 2019). In Firmicutes, the Clostridia includes various types of thermophilic, hydrogenogenic carboxydotrophs that harbor multiple *cooS* genes and feature the Wood–Ljungdahl pathway (WLP) for carbon fixation (Techtmann et al. 2012; Shin et al. 2016; Inoue et al. 2019).

The functions of these *cooS* genes have been predicted from their genomic contexts such as ECH, WLP, and ferredoxin–NAD(P)H oxidoreductase (Techtmann et al. 2012; Inoue et al. 2019), which are presumed to be regulated by CO-responsive transcription factors, such as CooA and RcoM (Shelver et al. 1995; Komori et al. 2007; Kerby et al. 2008). However, recent studies of two hydrogenogenic carboxydotrophs, *Carboxydothermus pertinax* and *Thermoanaerobacter kivui*, show that the enzymatic coupling of *cooS* and *ech* genes that are distantly encoded in their respective genomes enables CO-dependent H_2_ production (Fukuyama et al. 2018, 2019a; Schoelmerich and Müller 2019). Moreover, *cooS* expression is upregulated in the presence of CO, despite the fact that no sequence motif is recognized by the CO-responsive transcriptional activator CooA in *C. pertinax* (Fukuyama et al. 2018, 2019a). Similarly, *ech* expression is upregulated in the presence of CO, although the genome does not encode any previously described CO-responsive transcription factors in *T. kivui* (Schoelmerich and Müller 2019). These studies suggest that there are previously unknown transcriptional response mechanisms to CO. Therefore, the direct observation of transcriptional, proteomic, and metabolic changes under CO is required to understand the metabolisms of hydrogenogenic carboxydotrophic bacteria.

*Calderihabitans maritimus* KKC1 is a thermophilic, obligately anaerobic, hydrogenogenic, carboxydotrophic bacterium in the Clostridia, closely related to *Moorella*, and was isolated from a core sample taken from marine sediment in the Kikai Caldera, Japan (Yoneda et al. 2013). *C. maritimus* KKC1 can grow anaerobically on 100% CO while producing H_2_ and CO_2_ with or without additional electron acceptors such as thiosulfate, sulfite, ferric citrate, amorphous Fe(III) oxide, Fe_2_O_3_, or fumarate, and on organic compounds, such as pyruvate, in the presence of thiosulfate. *C. maritimus* KKC1 requires yeast extract for CO-dependent growth unlike chemolithoautotrophic, hydrogenogenic carboxydotrophs, such as *Moorella stamsii* and *Carboxydothermus hydrogenoformans*, and is unable to grow with H_2_ and CO_2_ unlike chemolithoautotrophic acetogens, such as *Moorella thermoacetica* (Svetlichny et al. 1991; Drake and Daniel 2004; Alves et al. 2013; Yoneda et al. 2013). The draft genome of *C. maritimus* KKC1 encodes six *cooS* genes that are categorized by their genomic contexts, as follows: *cooS1* (KKC1_RS04465), WLP; *cooS2* (KKC1_RS06675), ECH; *cooS3* (KKC1_RS06585), ferredoxin-NAD(P)H oxidoreductase; *cooS4* (KKC1_RS12505), a cysteine synthase and ABC transporter; *cooS5* (KKC1_RS04925), 2-oxoglutarate:ferredoxin oxidoreductase (Kor); and *cooS6* (KKC1_RS10495), CooA (Omae et al. 2017). These six *cooS* genes harbor the complete sequence motifs that form three types of metal clusters for catalysis, although *cooS1* is frame-shifted like other hydrogenogenic, carboxydotrophic *Moorella* and *Carboxydothermus* species possibly as a result of cultivation at high CO concentrations (Wu et al. 2005; Omae et al. 2017; Poehlein et al. 2018; Fukuyama et al. 2018, 2019b). To the best of our knowledge, the genome contains the highest number of *cooS* genes (Omae et al. 2017; Toshchakov et al. 2018) and encodes six hydrogenase gene clusters that include two *ech* gene clusters, a *coo*-type gene cluster (*ech1*, KKC1_RS06640–KKC1_RS06665) with *cooS2*, and a *hyc*/*hyf*-type gene cluster (*ech2*, KKC1_RS01155–KKC1_RS01200) with putative formate dehydrogenase genes (Omae et al. 2017). The genome only harbors one *cooA* gene as a CO-responsive transcription factor.

The multiplicity of Ni-CODHs and hydrogenases and the broad usage of electron acceptors in *C. maritimus* KKC1 suggest genomic adaptation for its carboxydotrophic growth (Yoneda et al. 2013; Omae et al. 2017); however, its metabolic and transcriptional responses to CO remain largely unknown. In this study, we performed a transcriptome analysis of *C. maritimus* KKC1 grown in the presence or absence of CO using RNA sequencing (RNA-seq). Under CO conditions, we found the evidence of transcriptional changes for Ni-CODHs and hydrogenases, and for anaerobic respiration, carbon and nitrogen metabolism, and transcription factors. We suggest that the transcriptional response mechanism to CO involves multiple transcription factors.

## Materials and methods

### Cultivation of *C. maritimus* KKC1

**Fig. 1:**
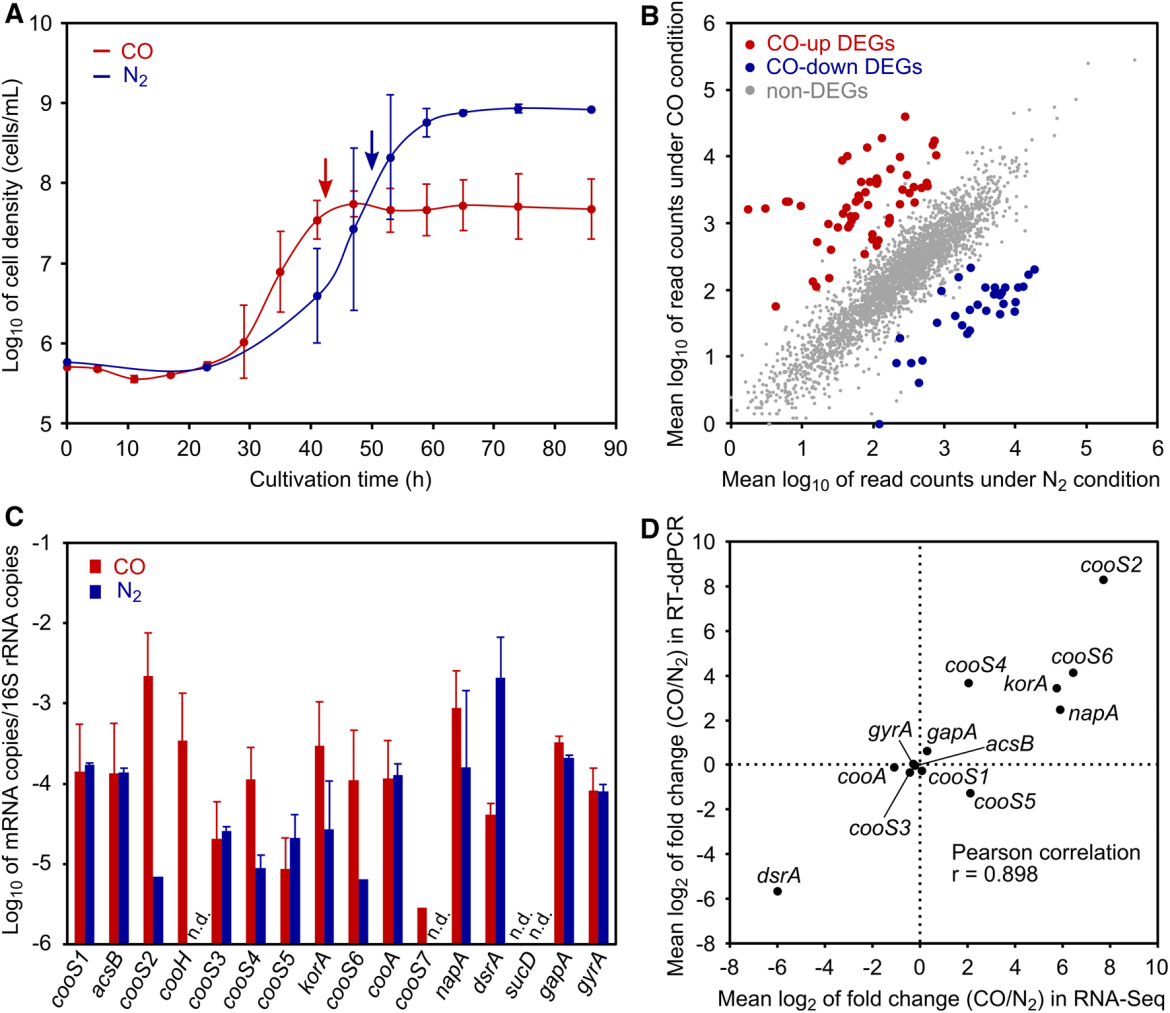
Overview of RNA-seq and RT-ddPCR datasets. **a** Growth curves of *C. maritimus* KKC1 in the presence (red) or absence (blue) of CO. The mean values of log10-transformed cell densities are plotted. Error bars indicate the standard deviation from three biological replicates. **b** Plots of read counts from genes in the presence or absence of CO. The mean values of log10-transformed read counts from two biological replicates are plotted. Upregulated DEGs in the presence of CO, downregulated DEGs, and non-DEGs are colored red, blue, and gray, respectively. **c** mRNA quantification by RT-ddPCR. The mean values of log10-transformed relative abundances of mRNA relative to 16S rRNA are shown. Values below the lower detection limit were omitted, although all samples were quantified with three biological replicates. Error bars indicate the standard deviation only when at least two values were above lower detection limit. **d** Comparison of fold-change values between RNA-seq and RT-ddPCR data. The mean values of log2-transformed fold changes of each gene are plotted. *n.d*. not detected

*Calderihabitans maritimus* KKC1 was cultured in modified NBRC 1251 medium, pH 7.5 at 65 °C under 100% CO or 100% N_2_ gas as previously described with slight modification (Yoneda et al. 2013). The medium contained 1 g sodium pyruvate, 1 g Na_2_S_2_O_3_·5H_2_O, 50 mg yeast extract, 16 g NaCl, 3.9 g MgSO_4 _·7H_2_O, 3.9 g MgCl_2_·6H_2_O, 0.14 g CaCl_2_·2H_2_O, 0.65 g KCl, 0.5 g NaHCO_3_, 0.1 g NH_4_Cl, 0.1 g KH_2_PO_4_, 0.1 g NaBr, 10 mg Na_2_SiO_3_, 30 mg H_3_BO_3_, 15 mg SrCl_2_·6H_2_O, 6.6 mg FeCl_3_, 0.05 mg KI, 0.05 mg NaNO_3_, 10 mL trace mineral solution SL-4 (Pfennig and Lippert 1966), 1.0 mL vitamin solution (Wolin et al. 1963), 0.5 mg resazurin, and 0.1 g Na_2_S·9H_2_O per liter. Cultivation was performed in 100 mL of medium in a 250-mL bottle sealed with a rubber stopper and a polypropylene screw cap. The cells grown under 100% N_2_ gas were inoculated at 10^5^–10^6^ cells/mL into the fresh media under both 100% CO and 100% N_2_ gas conditions with three biological replicates. Cell growth was determined using an S3e Cell Sorter (Bio-Rad, Hercules, CA, USA) by counting the number of fluorescent signals from SYBR Green I-stained cells. Consumption of CO and evolution of H_2_ were analyzed by a GC-2014 gas chromatography system (Shimadzu, Kyoto, Japan) equipped with a thermal conductivity detector and a Shincarbon ST packed column (Shinwa Chemical Industries, Kyoto, Japan) using argon as the carrier gas. For RNA extraction, cells were collected on a 0.22-μm Durapore membrane filter (Merck Millipore, Burlington, MA, USA) at the late-exponential phase (Fig. 1a), and then stored in RNA*later* RNA Stabilization Reagent (Qiagen, Hilden, Germany) at − 85 °C until RNA extraction.

### RNA isolation and cDNA synthesis

RNA isolation and cDNA synthesis were performed, as previously described (Fukuyama et al. 2018, 2019a). Total RNA was extracted using a mirVana miRNA Extraction Kit (Ambion, Austin, TX, USA), and the remaining genomic DNA was removed using TURBO DNase (Ambion). Total RNA was then purified using Agencourt RNAClean XP beads (Beckman Coulter, Brea, CA, USA), and quantified and quality controlled using a Qubit RNA HS Assay Kit (Invitrogen, Carlsbad, CA, USA) and an Agilent RNA 6000 Pico Kit on an Agilent 2100 Bioanalyzer (Agilent Technologies, Santa Clara, CA, USA). For RNA-seq analysis, rRNA was removed from the purified total RNA using a Ribo-Zero rRNA Removal Kit for Bacteria (Illumina, San Diego, CA, USA), and double-stranded cDNA was synthesized using a PrimeScript Double Strand cDNA Synthesis Kit (Takara, Shiga, Japan). For reverse transcriptase droplet digital polymerase chain reaction (RT-ddPCR), single-stranded cDNA was synthesized using a SuperScript III First-Strand Synthesis System (Invitrogen).

### RNA-seq and data analysis

A DNA library for RNA-seq was prepared using a Nextera XT DNA Library Prep Kit (Illumina) with two biological replicates. The library was quantified and quality controlled using KAPA Library Quantification Kits (KAPA Biosystems, Wilmington, MA, USA) on a Thermal Cycler Dice Real Time System Single (Takara) and an Agilent High Sensitivity DNA Kit on an Agilent 2100 Bioanalyzer (Agilent Technologies). Sequencing was performed on the Illumina MiSeq instrument with an MiSeq Reagent Kit v3 (150 cycles), which generated 15,716,607 paired-end reads.

Quality filtering was performed using an FASTX-Toolkit version 0.0.14 (https://hannonlab.cshl.edu/fastx_toolkit/), with the reads being Q30 for > 80% of the bases. Reads from the remaining rRNA were removed using a BLASTn search (Camacho et al. 2009). The filtered reads were mapped onto the draft genome sequence (GCF_002207765.1) of *C. maritimus* KKC1 obtained from the National Centre for Biotechnology Information (NCBI) assembly database (NCBI Resource Coordinators 2018) using HISAT2 version 2.1.0 (Kim et al. 2015). The mapped data were checked using SAMtools version 0.1.19 (Li et al. 2009), and mapped reads per gene were counted using featureCounts from the Subread package version 1.6.3 (Liao et al. 2014). Normalization and differential expression analysis were performed using the R package edgeR version 3.22.5 (Robinson et al. 2009). Differentially expressed genes (DEGs) were identified using the functions “glmQLFit” and “glmTreat” with a fold-change significantly greater than 1.5 at a false discovery rate of ≤ 0.001. The read processing and data set statistics for differential expression analysis are summarized in Tables S1 and S2, respectively.

Functional annotation of the DEGs was performed automatically using EggNOG-mapper version 1.0.3 in HMMER mapping mode (Huerta-Cepas et al. 2017) and RAST server version 2.0 with RASTtk pipeline (Brettin et al. 2015). Annotation was manually confirmed using a BLASTp search (Camacho et al. 2009). Pathway analysis was performed using the Kyoto Encyclopedia of Genes and Genomes mapper version 3.2 (Kanehisa et al. 2012). N-terminal signal sequences were predicted using SignalP server version 5.0 (Almagro Armenteros et al. 2019), and hydrogenase annotation was performed using HydDB server (Søndergaard et al. 2016).

### Prediction of transcriptional units and identification of transcription-factor-binding motifs

A unit of polycistronically transcribed genes, including DEGs, was manually predicted by checking the read alignments against the draft genome sequence of *C. maritimus* KKC1 using Integrative Genomics Viewer version 2.4.16 (Thorvaldsdóttir et al. 2013) (Table S3). The transcriptional unit comprised continuous same-stranded genes with continuous- and constant-read alignments. To discover sequence motifs in the upstream regions of DEGs, 300-bp upstream sequences from the start codon of the first gene in the transcriptional unit were curated from the genome sequence. Sequence motifs were searched using MEME version 4.12.0 (Bailey et al. 2009), and sequence logos were created using WebLogo version 2.8.2 (Crooks et al. 2004).

### RT-ddPCR

To validate the RNA-seq data, RT-ddPCR was performed using a QX200 Droplet Digital PCR System (Bio-Rad) with three biological replicates. Each ddPCR mixture comprised 10 μL of 2 × ddPCR EvaGreen Supermix (Bio-Rad), 0.4 μL of 5 μM primer mix, and 1 μL of appropriately diluted cDNA in a final volume of 20 μL. Droplets were generated using a Droplet Generator (Bio-Rad) with 70 μL of Droplet Generator Oil for EvaGreen (Bio-Rad), and then transferred to a 96-well PCR plate (Eppendorf, Hamburg, Germany) and heat sealed. PCR amplification was performed on a TaKaRa PCR Thermal Cycler Dice *Touch* at 95 °C for 10 min followed by 40 cycles at 94 °C for 30 s and 58 °C for 60 s, one cycle at 98 °C for 10 min, and ending at 8 °C. After amplification, fluorescent signals from the droplets in each well were automatically measured using a Droplet Reader (Bio-Rad).

ddPCR data were analyzed using QuantaSoft software version 1.7.4 (Bio-Rad). To separate positive and negative droplets, thresholds of the fluorescent signals were manually set. The number of target DNA molecules in the reaction mixture was determined from the ratio of positive to total droplets. Data were quality controlled with > 10,000 total droplets in each sample and < 10 positive droplets in the negative control, except for the *korA* primers (< 20 positive droplets in the negative control). The lower limit of detection was set at > 10 positive droplets, except for the *korA* primers (> 20 positive droplets). To compare expression levels in different samples, concentrations of the target genes were normalized against the concentrations of 16S rRNA. All primers used for ddPCR were quality controlled with the lengths and melting curves of the products checked by agarose gel electrophoresis and real-time PCR, respectively (Table S4).

## Data availability

The raw reads for RNA-Seq and the gene-expression profiles have been deposited in the NCBI/ENA/DDBJ Sequence Read Archive under accession number DRA008406.

## Results

### Overview of RNA-seq analysis

We performed RNA-seq experiments for late-exponential cultures of *C. maritimus* KKC1 grown under 100% CO and N_2_ gas conditions in biological duplicates (Fig. 1a). To enable growth under N_2_ conditions, pyruvate and thiosulfate were added to the medium under both gas conditions. Under CO conditions, conversion of ~ 10% of CO to H_2_ was observed at the sampling point by gas chromatography (data not shown). Over 3 million high-quality RNA-seq reads in each sample were mapped with sufficient coverages (> 100 × mean coverage in each sample) and mapping efficiencies (> 80% in each sample) (Table S1). The biological replicates for the CO and N_2_ conditions of the RNA-seq experiments were highly reproducible with Pearson’s correlation coefficients of 0.968 and 0.970, respectively (Fig. S1).

Normalization and differential gene-expression analysis were performed using mapped RNA-seq data. Of the 3,114 genes in *C. maritimus* KKC1, 90 were differentially expressed between the two conditions, with these DEGs including 58 upregulated and 32 downregulated genes in the presence of CO and log_2_-fold changes for CO/N_2_ ranging from 2.57 to 10.3 and from − 7.16 to − 2.68, respectively (Fig. 1b and Table S2). Approximately 60% of the upregulated and ~ 70% of the downregulated DEGs in the presence of CO were grouped into the “metabolism” categories of Clusters of Orthologous Groups (COGs) (Galperin et al. 2015), with > 40% of both upregulated and downregulated DEGs were grouped in the COG functional category “C” (energy production and conversion) (Table S2). These data are comparable with those obtained by a recent RNA-seq study of *C. pertinax* (Fukuyama et al. 2019a).

The expression levels of the seven DEGs and eight non-DEGs, including *cooS* genes (*cooS1* through *cooS6*) and house-keeping genes (*gapA* and *gyrA*), were confirmed by RT-ddPCR (Fig. 1c). The *C. maritimus* KKC1genome also contains two partial *cooS*-like gene fragments (KKC1_RS14835 and KKC1_RS01100) (Omae et al. 2017) sharing 65 bp and 16 aa perfect matches encoding one protein (hereafter designated as CooS7). The expression level of *cooS7* judged as non-DEGs was also tested. The fold-change values in RNA-seq and RT-ddPCR data were highly correlated, with a Pearson’s correlation coefficient of 0.898 (Fig. 1d), suggesting that our RNA-seq data captured the precise transcriptional changes between CO and N_2_ conditions.

### CO-dependent differential expression of multiple genes encoding Ni-CODHs and hydrogenases

**Fig. 2:**
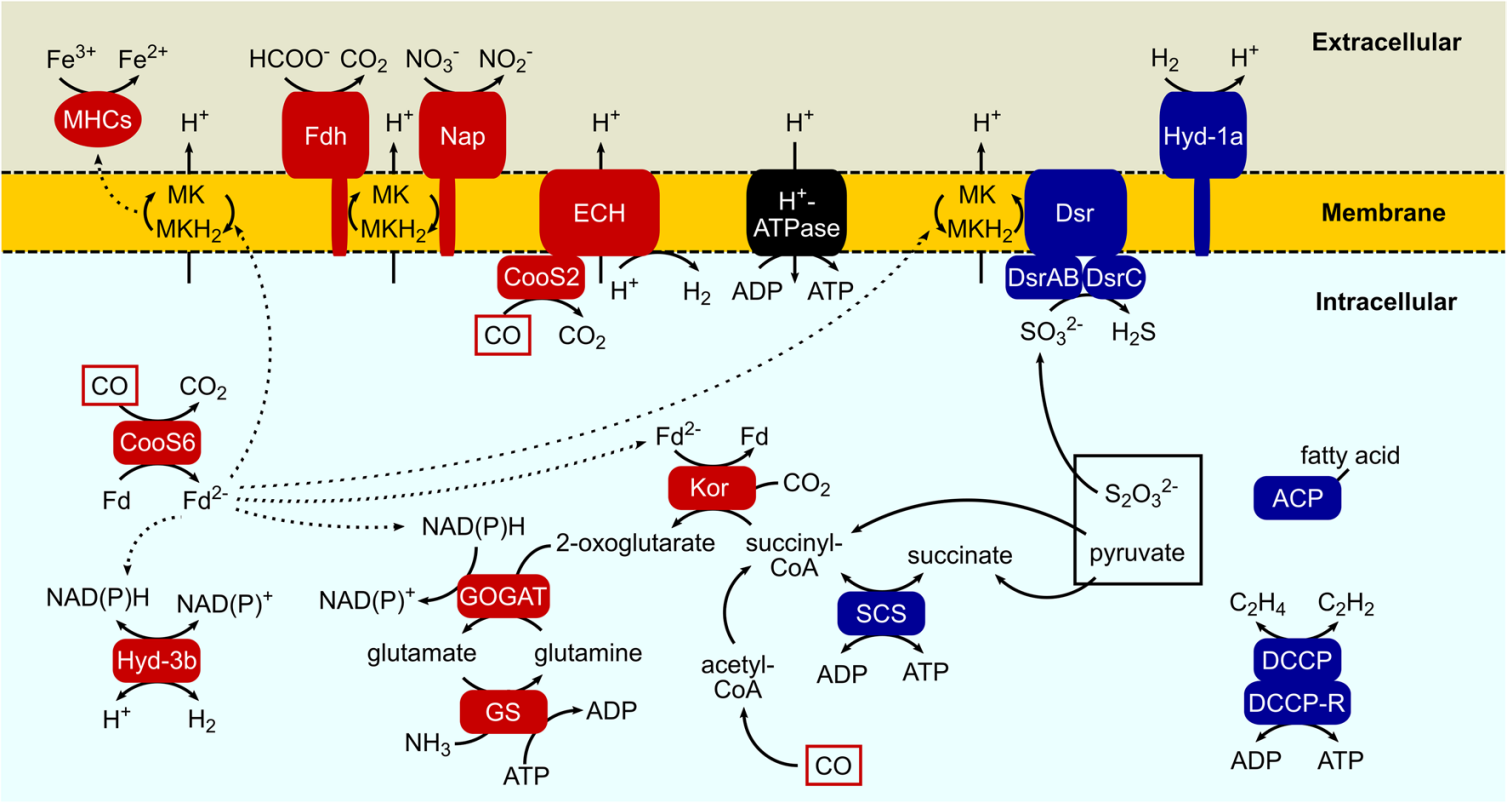
Schematic representation of metabolic pathways in *C. maritimus* KKC1 with DEGs. Only pathways discussed in the text are shown. Protein machineries with upregulated and downregulated DEGs in the presence of CO are colored red and blue, respectively. ATP synthase (H^+^-ATPase) is colored black. Dotted arrows indicate the possible pathways of electron flow from CO oxidation. *MK* menaquinone, *MKH*_2_ menaquinol

We first focused on transcriptional changes in Ni-CODHs and hydrogenases, which are responsible for carboxydotrophic and hydrogenogenic growth. Of the seven *cooS* genes in *C. maritimus* KKC1, only *cooS2* and *cooS6* were identified as upregulated DEGs in the presence of CO (Fig. 2; Tables 1 and S2). All of the genes in the *cooS2*–*ech1* (*coo*-type) gene cluster (KKC1_RS06640–KKC1_RS06680) were upregulated DEGs in the presence of CO, indicating that the Ni-CODH/ECH (Coo-type) complex was responsible for CO-dependent H_2_ production, similar to other hydrogenogenic carboxydotrophs (Soboh et al. 2002; Singer et al. 2006; Schut et al. 2016; Schoelmerich and Müller 2019). Conversely, the expression level of the *ech2* (*hyc*/*hyf*-type) gene cluster (KKC1_RS01155–KKC1_RS01200) was unchanged under CO conditions, indicating a potential function with its gene neighbor encoding the putative formate dehydrogenase.

**Table 1:** List of Ni-CODHs and hydrogenases identified in DEGs

Functional group	Locus tag	Log_2_FC	Annotation	COG number (category)^*a*^
CooS2/Ech1	KKC1_RS06640	8.4	CooM	COG0651,COG1007 (C)
CooS2/Ech1	KKC1_RS06645	7.9	CooK	COG0650 (C)
CooS2/Ech1	KKC1_RS06650	9.5	CooL	COG3260 (C)
CooS2/Ech1	KKC1_RS06655	8.8	CooX	COG1143 (C)
CooS2/Ech1	KKC1_RS06660	10.3	CooU	(C)
CooS2/Ech1	KKC1_RS06665	9.0	CooH	COG3261 (C)
CooS2/Ech1	KKC1_RS06670	8.5	CooF	COG1142 (C)
CooS2/Ech1	KKC1_RS06675	7.7	CooS	COG1151 (C)
CooS2/Ech1	KKC1_RS06680	6.0	CooC	COG3640 (D)
CooS6	KKC1_RS10495	6.5	CooS	COG1151 (C)
Hyd-3b	KKC1_RS10615	5.0	Group 3b Ni,Fe-hydrogenase beta subunit	COG1145 (C)
Hyd-3b	KKC1_RS10620	5.0	Group 3b Ni,Fe-hydrogenase gamma subunit	COG0543 (C)
Hyd-3b	KKC1_RS10625	4.8	Group 3b Ni,Fe-hydrogenase small subunit	COG1941 (C)
Hyd-3b	KKC1_RS10630	5.8	Group 3b Ni,Fe-hydrogenase large subunit	COG3259 (C)
Hyd-3b	KKC1_RS10635	4.8	Ni,Fe-hydrogenase maturation factor	(O)
Hyd-1a	KKC1_RS00385	− 5.2	Group 1a Ni,Fe-hydrogenase small subunit	COG1740 (C)
Hyd-1a	KKC1_RS00390	− 4.1	Group 1a Ni,Fe-hydrogenase large subunit	COG0374 (C)
Hyd-1a	KKC1_RS00395	− 4.8	Group 1a Ni,Fe-hydrogenase cytochrome *b* subunit	COG1969 (C)
Hyd-1a	KKC1_RS00400	− 4.1	Ni,Fe-hydrogenase maturation factor	COG0680 (O)

^*a*^COG number and category were annotated by EggNOG mapper. Note that some COG categories were annotated, even when COG numbers were not annotated

*FC* fold change

*cooS6* expression was upregulated, whereas that of an adjacent upstream gene (*cooA*) was unchanged, and the other five *cooS* genes were not identified as DEGs (Figs. 1d and 2; Table 1 and S2). It should be noted that the expression level of *cooS4* was higher in the presence of CO in both RNA-Seq and RT-ddPCR experiments albeit a non-DEG (Fig. 1c and d; Table S2). RNA-Seq and RT-ddPCR revealed that *cooS1* and *acsB*, which are involved in WLP, were highly expressed in the presence and absence of CO (although *cooS1* is frame-shifted) (Fig. 1c and Table S2), similar to results from recent RNA-seq data for *C. pertinax* (Fukuyama et al. 2019a). These data suggested that gene transcription for WLP is unchanged between CO and N_2_ conditions in these hydrogenogenic, carboxydotrophic bacteria.

Two of the four putative [NiFe] hydrogenase genes other than the two *ech* genes in *C. maritimus* KKC1 were identified as DEGs (Fig. 2; Table 1 and S2). Under CO conditions, the expression levels of *hyd-3b* genes (KKC1_RS10620–KKC1_RS10635) encoding group 3b [NiFe] hydrogenase catalytic subunits and their maturation protease were upregulated, whereas those of *hyd-1a* genes (KKC1_RS00385–KKC1_RS00400) encoding group 1a [NiFe] hydrogenase catalytic subunits, a cytochrome *b* subunit, and their maturation protease were downregulated. The group 3b [NiFe] hydrogenase is a cytoplasmic, bidirectional hydrogenase that directly couples NADPH oxidation to H_2_ evolution (Greening et al. 2016; Søndergaard et al. 2016), although it remains unclear whether NADPH is utilized by this enzyme in *C. maritimus* KKC1. Conversely, the group 1a [NiFe] hydrogenase is a periplasmic, unidirectional H_2_-uptake hydrogenase, and the cytochrome *b* subunit is considered a putative electron carrier, as are other group 1 hydrogenases (Greening et al. 2016; Søndergaard et al. 2016); however, a terminal electron acceptor has not been identified in *C. maritimus* KKC1.

### Transcriptional changes in anaerobic respiration systems under CO conditions

Other than Ni-CODHs and hydrogenases, anaerobic respiration genes required for energy production and conversion were identified as DEGs. In the presence of CO, the expression levels of gene clusters encoding periplasmic formate dehydrogenase (Fdh)-like proteins (KKC1_RS05300–KKC1_RS05315) and periplasmic nitrate reductase (Nap)-like proteins (KKC1_RS14135–KKC1_RS14170) were significantly upregulated, whereas those of a gene cluster encoding dissimilatory sulfite reductase (Dsr)-like proteins (KKC1_RS04115–KKC1_RS04185 and KKC1_RS04195) were downregulated (Fig. 2; Table 2 and S2). The Dsr complex couples sulfite reduction with a proton-translocating menaquinone cycle (Venceslau et al. 2014; Santos et al. 2015; Anantharaman et al. 2018), whereas Fdh and Nap couple formate oxidation with nitrate reduction to drive the menaquinone cycle (Fig. 2) (Richardson et al. 2004; Cerqueira et al. 2015). Under N_2_ conditions, energy production is mainly performed through pyruvate oxidation and thiosulfate reduction (Yoneda et al. 2013). Our data suggested that the Dsr complex catalyzes electron transfer to the menaquinone pool by reducing the sulfite derived from thiosulfate in the absence of CO. Conversely, in the presence of CO, both Fdh- and Nap-like proteins would maintain the menaquinone cycle instead of Dsr, although authentic substrates of these proteins have not been identified.

**Table 2:** List of anaerobic respiration machineries identified in DEGs

Functional group	Locus tag	Log_2_FC	Annotation	COG number (category)^*a*^
Fdh	KKC1_RS05300	3.1	Twin-arginine translocase TatA/TatE family subunit	(U)
Fdh	KKC1_RS05305	3.7	FdoI	COG2864 (C)
Fdh	KKC1_RS05310	3.4	FdoH	COG0437 (C)
Fdh	KKC1_RS05315	3.7	FdoG	COG0243 (C)
Nap	KKC1_RS14135	5.8	Twin-arginine translocase TatA/TatE family subunit	(U)
Nap	KKC1_RS14140	5.8	NapD	
Nap	KKC1_RS14145	5.7	NapB	
Nap	KKC1_RS14150	5.9	NapG	COG1145 (C)
Nap	KKC1_RS14155	5.9	NapA	COG0243 (C)
Nap	KKC1_RS14160	5.7	Hypothetical protein (pseudogene)	
Nap	KKC1_RS14165	5.8	NapH	COG0348 (C)
Nap	KKC1_RS14170	5.8	NapG	COG1145 (C)
MHCs	KKC1_RS04440	7.7	MHC-1 (12 × CX_2_H motifs)	(S)
MHCs	KKC1_RS08310	7.9	MHC-2 (7 × CX_2_H motifs)	(S)
MHCs	KKC1_RS11980	3.2	MHC-3 (12 × CX_2_H motifs)	(C)
Dsr	KKC1_RS04115	– 6.0	DsrT	(S)
Dsr	KKC1_RS04120	– 5.7	DsrK	
Dsr	KKC1_RS04125	– 6.1	DsrJ	(C)
Dsr	KKC1_RS04130	– 5.0	DsrO	COG0437 (C)
Dsr	KKC1_RS04135	– 5.8	DsrP	COG5557 (C)
Dsr	KKC1_RS04140	– 5.5	DsrM	COG2181 (C)
Dsr	KKC1_RS04145	– 6.0	DsrA	COG2221 (C)
Dsr	KKC1_RS04150	– 6.1	DsrB	COG2221 (C)
Dsr	KKC1_RS04155	– 6.7	DsrD	(S)
Dsr	KKC1_RS04160	– 6.6	Ferredoxin	(C)
Dsr	KKC1_RS04165	– 7.2	DsrC	COG2920 (P)
Dsr	KKC1_RS04170	– 5.1	Pyridine nucleotide-disulphide oxidoreductase	COG0446,COG0607 (P)
Dsr	KKC1_RS04175	– 5.3	DsrN	COG1797 (H)
Dsr	KKC1_RS04180	– 6.2	DsrM	COG2181 (C)
Dsr	KKC1_RS04185	– 6.1	DsrK	COG0247 (C)
Dsr	KKC1_RS04195	– 2.8	DsrE	COG1416 (S)

^*a*^COG number and category were annotated by EggNOG mapper. Note that some COG categories were annotated, even when COG numbers were not annotated

*FC* fold change

Additionally, we found that the upregulated DEGs included three genes encoding the putative extracellular multiheme cytochromes *c* (MHCs; *mhc-1*, *mhc-2*, and *mhc-3*, corresponding to KKC1_RS04440, KKC1_RS08310, and KKC1_RS11980, respectively), which are responsible for redox cycles of metals including Fe(III), nitrogen compounds, and sulfur compounds by catalyzing electron transfer from/to quinone pools in the extracellular space (Mowat and Chapman 2005; Zhong and Shi 2018; Chong et al. 2018) (Fig. 2; Table 2 and S2). MHC-1, MHC-2, and MHC-3 have 12, 7, and 12 CX_2_CH motifs capable of binding a heme moiety, respectively, and all three proteins were predicted to have N-terminal signal sequences for extracellular localization, indicating their involvement in extracellular electron transfer.

### Carbon and nitrogen metabolism as a possible redox-buffering system in the CO response

Reducing equivalents from CO can be utilized for other redox metabolic pathways. In the presence of CO, genes encoding Kor-like proteins (KKC1_RS04895–KKC1_RS04910), a glutamine synthetase (GS)-like protein (KKC1_RS05230), and glutamate synthase (GOGAT)-like proteins (KKC1_RS05235–KKC1_RS05240) were identified as upregulated DEGs (Fig. 2; Table 3 and S2). Kor catalyzes the reversible conversion of succinyl-CoA to 2-oxoglutarate through (de)carboxylating reactions, using ferredoxin as an electron carrier (Fig. 2) (Yamamoto et al. 2010; Li and Elliott 2016; Chen et al. 2019). GOGAT produces two molecules of glutamate from 2-oxoglutarate and glutamine using NAD(P)H (Vanoni and Curti 2005), whereas GS produces glutamine from glutamate and ammonia using ATP (Eisenberg et al. 2000). It should be noted that a gene encoding a succinyl-CoA synthetase (SCS) μ subunit (KKC1_RS08680) was significantly downregulated in the presence of CO (Fig. 2; Table 3 and S2); however, *C. maritimus* KKC1 possesses three SCS gene sets, and the expression levels of the other two SCS genes were unchanged under CO conditions (Table S2).

**Table 3:** List of DEGs related to carbon and nitrogen metabolisms

Functional group	Locus tag	Log_2_FC	Annotation	COG number (category)^*a*^
Kor	KKC1_RS04895	6.0	KorD	(C)
Kor	KKC1_RS04900	5.8	KorA	COG0674 (C)
Kor	KKC1_RS04905	5.8	KorB	COG1013 (C)
Kor	KKC1_RS04910	5.0	KorG	COG1014 (C)
GS/GOGAT	KKC1_RS05230	3.8	Glutamine synthetase	COG0174 (E)
GS/GOGAT	KKC1_RS05235	4.3	Glutamate synthase domain 1	COG0067 (E)
GS/GOGAT	KKC1_RS05240	3.3	Glutamate synthase domain 2	COG0069 (E)
SCS	KKC1_RS08680	− 6.4	Succinyl-CoA synthetase subunit alpha	COG0074 (C)
ACP	KKC1_RS01055	− 2.7	Acyl carrier protein	COG0236 (I)
DCCP	KKC1_RS14820	− 5.5	DCCP	COG1775 (E)
DCCP	KKC1_RS14825	− 4.5	DCCP reductase	COG1924 (I)

^*a*^COG number and category were annotated by EggNOG mapper. Note that some COG categories were annotated, even when COG numbers were not annotated

*FC* fold change

Genes for a double-cubane cluster protein (DCCP), DCCP reductase (KKC1_RS14820–KKC1_RS14825), and an acyl carrier protein (KKC1_RS01055) were also identified as downregulated DEGs under CO conditions (Fig. 2; Table 3 and S2). DCCP and DCCP reductase represent a recently identified, novel, ATP-dependent oxidoreductase system in *C. hydrogenoformans* (Jeoung and Dobbek 2018) and the amino acid identities of DCCP and DCCP reductase homologs of *C. maritimus* KKC1 to those of *C. hydrogenoformans* were 57% and 41%, respectively. DCCP and DCCP reductase reduce acetylene to ethylene, with this activity inhibited by CO. Additionally, the acyl carrier protein plays a central role in fatty-acid biosynthesis, which requires the NADPH-dependent reduction of an acyl chain (Chan and Vogel 2010).

### Transcription factors as DEGs

**Fig. 3:**
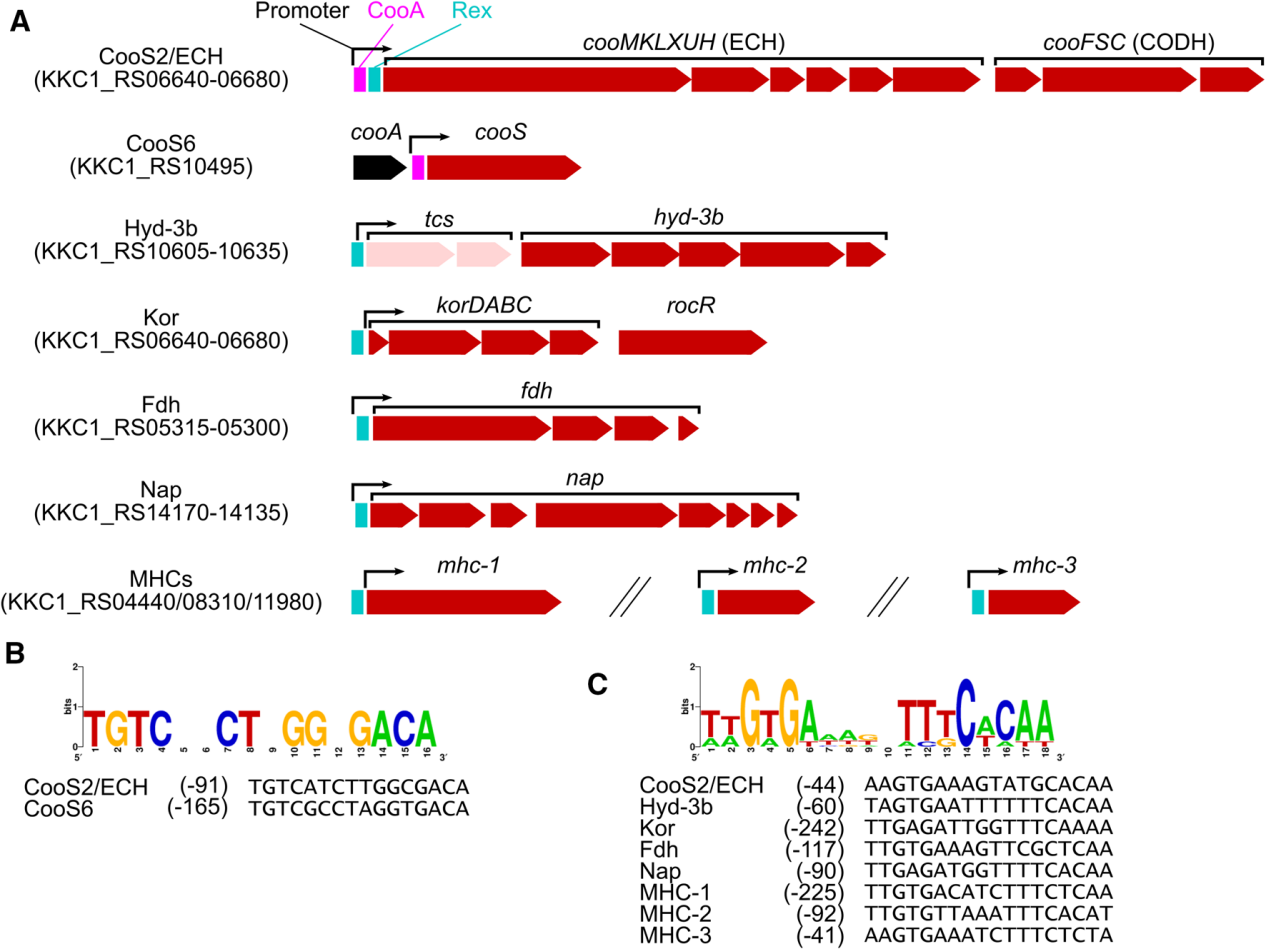
Prediction of transcriptional response to CO in *C. maritimus* KKC1. **a** Predicted structure of CO-responsive transcriptional units regulated by CooA and Rex. The predicted promoter regions (− 10 and − 35 sequences) are indicated by arrows. Regions of sequence motifs recognized by CooA and Rex are colored magenta and cyan, respectively. Upregulated DEGs in the presence of CO and upregulated non-DEGs are colored red and pale red, respectively. The *cooA* gene, which is upstream of *cooS6*, is shown in black. *tcs*, two-component system genes. **b**,**c** Putative **b** CooA- and **c** Rex-binding sequence motifs. The sequence logo is shown above each sequence alignment, and the number of bases from the start codon is shown in parentheses

Putative transcription factors were also identified among the DEGs. Genes encoding two RocR-like AAA^+^ superfamily transcriptional activators (KKC1_RS02905 and KKC1_RS04915) and one TetR/AcrR family transcriptional regulator (KKC1_RS07140) were upregulated in the presence of CO, whereas an LysR family transcriptional regulator (KKC1_RS14830) was downregulated (Table 4 and S2). To analyze the relationships between these transcription factors and their targets, the transcriptional units were predicted using the mapped RNA-seq data that included DEGs (Table S3). One gene encoding an RocR-like protein (KKC1_RS04915) was adjacent to the *kor* genes, which were significantly upregulated, whereas the other gene (KKC1_RS02905) was an orphan. These two proteins shared 89% amino acid identity and possessed a conserved HX_2_CX_2_CX_5_C motif, possibly used for metal binding in the N-terminal sensor domain. RocR-like proteins were originally positive regulators of arginine catabolism in bacteria (Calogero et al. 1994). Genes encoding a TetR/AcrR-like protein and a LysR-like protein were located at transcriptional units encoding efflux transporter systems and DCCP/DCCP reductase, respectively (Tables S2 and S3). Moreover, our prediction of the transcriptional units revealed that two-component-system-like genes (KKC1_RS10605–KKC1_RS10610) were located in the unit containing *hyd-3b* genes, although these two-component-system-like genes were not identified as DEGs (Fig. 3a; Tables S2 and S3). These transcription factors might alter DEG expression levels in *C. maritimus* KKC1.

**Table 4:** List of transcription factors in DEGs

Functional group	Locus tag	Log_2_FC	Annotation	COG number (category)^*a*^
RocR	KKC1_RS02905	3.3	RocR-like transcriptional activator	COG3829 (K)
RocR	KKC1_RS04915	4.7	RocR-like transcriptional activator	COG3829 (K)
TetR	KKC1_RS07140	2.8	TetR/AcrR family transcriptional regulator	(K)
LysR	KKC1_RS14830	− 2.9	LysR family transcriptional regulator	COG0583 (K)

^*a*^COG number and category were annotated by EggNOG mapper. Note that some COG categories were annotated, even when COG numbers were not annotated

*FC *fold change

### Transcription factor-binding motifs

To identify transcription-factor-binding motifs in the upstream regions of the DEGs, we searched for palindromic sequence motifs in the predicted transcriptional units (Table S3). Putative sequence motifs recognized by a CO-sensing transcriptional activator CooA and a redox-sensing transcriptional regulator Rex were identified in the transcriptional units upregulated by CO, whereas no transcription-factor-binding motif was identified in the downregulated transcriptional units (Fig. 3). A CooA-binding consensus sequence (5′-TGTC-N_8_-GACA) was found in the upstream regions of the *cooS2*–*ech1* gene cluster and *cooS6*, as previously reported (Fig. 3a and b) (Omae et al. 2017), whereas Rex-binding sequence (5′-TTGTGA-N_6_-TCACAA)-like motifs were found in the upstream regions of the transcriptional units containing the *cooS2*–*ech1*, *hyd-3b*, *kor*, *fdh*, and *nap* gene clusters and three *mhc* genes (Fig. 3a and c). These data implied that CooA and Rex are involved in the upregulation of these genes in the presence of CO in situations involving direct CO sensing and redox sensing, respectively.

## Discussion

We examined transcriptomic changes in *C. maritimus* KKC1 growing in the presence or absence of CO using RNA-seq analysis. Our data showed that of the seven *cooS* genes and six hydrogenase genes studied, the *cooS2*–*ech1* (*coo*-type) gene cluster (not the *hyc*/*hyf*-type gene cluster), the “orphan” *cooS6* gene, and bidirectional *hyd-3b* genes were upregulated under CO condition (Fig. 2 and Table 1). In hydrogenogenic carboxydotrophic *Moorella* species, genes for the *hyc*/*hyf*-type hydrogenase form a gene cluster with *cooS* and are responsible for CO-dependent H_2_ production (Poehlein et al. 2018; Fukuyama et al. 2019b), while in *Carboxydothermus* species, the *coo*-type works with *cooS* (Wu et al. 2005; Fukuyama et al. 2019a), suggesting that *C. maritimus* KKC1 utilizes a strategy similar to *Carboxydothermus* species rather than *Moorella* species for CO-dependent energy conservation. Upregulation of the “orphan” *cooS6* gene under CO conditions could induce the reduction of the ferredoxin pool and perturbation of cellular redox balance in *C. maritimus* KKC1. The bidirectional *hyd-3b* genes might balance such redox perturbation. Moreover, unlike *C. pertinax*, H_2_-uptake *hyd-1a* genes were downregulated under CO conditions in *C. maritimus* KKC1 (Fig. 2 and Table 1) (Fukuyama et al. 2019a). These data suggest that an H_2_-evolution system is predominant in the presence of CO because of the excessive reducing equivalents from CO in *C. maritimus* KKC1.

Our data support the broad usage of electron acceptors in anaerobic respiration of *C. maritimus* KKC1 (Fig. 2 and Table 2). We found that three *mhc* genes presumed to utilize Fe(III) were upregulated in the presence of CO. In *Thermincola potens*, MHCs are responsible for respiratory electron transfer to Fe(III) (Carlson et al. 2012), and genomic analysis of *Carboxydocella thermoautotrophica* indicated the possible involvement of MHCs in CO-dependent Fe(III) reduction (Toshchakov et al. 2018). Similar to these hydrogenogenic carboxydotrophs, *C. maritimus* KKC1 produces Fe(II) with ferric citrate, amorphous Fe(III) oxide, and Fe_2_O_3_ in the presence of CO, suggesting that Fe(III) is used as an electron acceptor under CO conditions (Yoneda et al. 2013). Therefore, these MHCs might be responsible for extracellular electron transfer to Fe(III) in *C. maritimus* KKC1. On the contrary, *dsr* genes that utilize sulfite in thiosulfate respiration were downregulated in the presence of CO although *C. maritimus* KKC1 is considered to couple thiosulfate reduction with CO or pyruvate oxidation (Yoneda et al. 2013). Thiosulfate respiration is conducted by a concerted way of thiosulfate/polysulfide reductase (Phs) and Dsr (Stoffels et al. 2011; Venceslau et al. 2014). We could not identify any *phs* genes in *C. maritimus* KKC1; therefore, a complete thiosulfate reduction pathway in this organism remains unknown. *C. pertinax,* which can also utilize Fe(III) and thiosulfate with CO or pyruvate oxidation, shows that expressions of the *mhc*-like genes, the *dsr* gene cluster (cpu_17430–17,360), and the *phs* gene cluster (cpu_06910–06,930) remain unchanged between CO and N_2_ conditions (Fukuyama et al. 2019a), suggesting that transcriptional responses in the anaerobic respiration pathway upon CO would differ between these two hydrogenogenic carboxydotrophs.

Oxidoreductase-like genes encoding Kor, GOGAT, and GS involved in carbon and nitrogen metabolisms were also upregulated under CO conditions in *C. maritimus* KKC1, strongly suggesting metabolic adaptation to cellular redox change upon CO oxidation (Fig. 2 and Table 3). Moreover, *kor* genes are upregulated in *Thermococcus onnurineus* under CO conditions, suggesting the possible involvement of Kor in carbon fixation (Moon et al. 2012), whereas the GS/GOGAT cycle is a redox-buffering system involving the consumption of NAD(P)H in *Caldicellulosiruptor bescii* and *Clostridium thermocellum* (Sander et al. 2015, 2019). It is possible that the enzymatic cycle involving Kor, GOGAT, and GS would assimilate carbon and nitrogen using excessive reducing equivalents from CO.

Additionally, we identified putative transcription factors as DEGs (Table 4) and putative CooA- and Rex-binding motifs in the upstream regions of upregulated DEGs (Fig. 3). As noted, the expression of *cooA* remained unchanged under CO conditions, suggesting that the basal expression level of *cooA* would be adequate to adapt to CO (Table S2). This phenomenon is different in *C. pertinax* and *Desulfovibrio vulgaris*, where *cooA* genes are upregulated in the presence of CO (Rajeev et al. 2012; Fukuyama et al. 2019a). Moreover, Rex is a redox-sensing transcriptional repressor and conserved among various bacteria, regardless of whether they are anaerobes or aerobes, and conformational changes from NAD^+^- to NADH-bound states induce transcription by releasing Rex from its recognition sequence (McLaughlin et al. 2010; Ravcheev et al. 2012). *C. maritimus* KKC1 possesses one Rex homolog (KKC1_RS10865), which exhibits 39% and 41% amino acid identities with those from *C. bescii* and *Clostridium acetobutylicum*, respectively. As described here, *C. bescii* Rex regulates various types of hydrogenases including ECH, and pyruvate:ferredoxin oxidoreductase belonging to the same family as Kor (Sander et al. 2019). In *Clostridium* species, Rex regulates the expressions of genes encoding various types of oxidoreductase, including nitrate reductases, hydrogenases, and WLP enzymes (Zhang et al. 2014). Therefore, cellular redox changes under CO conditions in *C. maritimus* KKC1 might result in a high NADH/NAD^+^ ratio to drive Rex-dependent transcriptional activation of oxidoreductase-like genes.

These findings suggest a possible multi-step transcriptional response to CO in *C. maritimus* KKC1 as follow: 1) upon CO exposure, CO-sensing CooA activates transcription of the *cooS2*–*ech1* gene cluster and *cooS6*, and these two Ni-CODHs catalyze CO oxidation to supply reducing equivalents within the cell; 2) excessive reducing equivalents from CO result in a high NADH/NAD^+^ ratio, followed by Rex-dependent transcriptional activation of the *cooS2*–*ech1*, *hyd-3b*, *kor*, *fdh,* and *nap* gene clusters, and three *mhc* genes; and 3) changes in the metabolisms or expression of transcription factors induce alterations in the transcription of other DEGs.

Our data highlight the diversity of CO-responsive transcriptional regulation in thermophilic, hydrogenogenic, carboxydotrophic bacteria. In *C. pertinax*, of the nine gene clusters upregulated in the presence of CO, including *cooS*, only the *ech* gene cluster is directly regulated by CooA (Fukuyama et al. 2019a), whereas the Rex-binding motif or those of other transcription factors are not found in upstream regions of these nine gene clusters. Additionally, in hydrogenogenic, carboxydotrophic *Moorella* strains, whose genomes encode no known CO-sensing transcription factor homologs, genes for RocR-like transcriptional activators (MOST_RS16225 in *M. stamsii* and MOTE_RS04420 in *M. thermoacetica* DSM 21,394) are located in the upstream regions of their Ni-CODH–ECH gene clusters. Because two genes for RocR-like proteins were upregulated under CO condition in *C. maritimus* KKC1 (Table 4), RocR-like proteins related to Ni-CODH–ECH gene clusters in these two *Moorella* species might be involved in response to CO. Moreover, a recent comparative genomics study of *Parageobacillus thermoglucosidasius*, a hydrogenogenic carboxydotroph also lacking known CO-sensing transcription factors, has found a transition-state regulator Hpr-binding sequence in the upstream region of its Ni-CODH–ECH gene cluster (Mohr et al. 2018). These findings imply previously undescribed transcriptional response mechanisms to CO. There could be various ways to respond to CO, including directly sensing CO, via stress caused by CO, or through cellular redox or metabolic changes via CO oxidation. Therefore, the diverse strategies for adaptation to CO-dependent metabolism would have been evolved in thermophilic, hydrogenogenic, carboxydotrophic bacteria.

## Electronic supplementary material

Below is the link to the electronic supplementary material.

Supplementary file1 (DOCX 303 kb)

Supplementary file2 (XLSX 778 kb)

## Abbreviations

CODH: Carbon monoxide dehydrogenase
Ni-CODH: Anaerobic Ni-containing CODH
ECH: Energy converting hydrogenase
WLP: Wood–Ljungdahl pathway
Kor: 2-Oxoglutarate:ferredoxin oxidoreductase
RNA-seq: RNA sequencing
RT-ddPCR: Reverse transcriptase droplet digital polymerase chain reaction
NCBI: The National Centre for Biotechnology Information
DEG: Differentially expressed gene
COG: Cluster of orthologous groups
Fdh: Periplasmic formate dehydrogenase
Nap: Periplasmic nitrate reductase
Dsr: Dissimilatory sulfite reductase
MHC: Extracellular multiheme cytochromes *c*
GS: Glutamine synthetase
GOGAT: Glutamate synthase
SCS: Succinyl-CoA synthetase
DCCP: Double-cubane cluster protein
