# Supplementary material for: Carbon monoxide-dependent transcriptional changes in a thermophilic, carbon monoxide-utilizing, hydrogen-evolving bacterium Calderihabitans maritimus KKC1 revealed by transcriptomic analysis
Source: Extremophiles. 2020 May 9;24(4):551–64. doi: 10.1007/s00792-020-01175-z (PMC7306483; doi:10.1007/s00792-020-01175-z)
Supplement: Supplementary file 1 — Supplementary file1 (DOCX 303 kb) [file 792_2020_1175_MOESM1_ESM.docx]

**Supplementary Material**

**Carbon Monoxide-dependent Transcriptional Changes in a Thermophilic, Carbon Monoxide-utilizing, Hydrogen-evolving Bacterium *Calderihabitans maritimus* KKC1 Revealed by Transcriptomic Analysis**

Masao Inoue, Hikaru Izumihara, Yuto Fukuyama, Kimiho Omae, Takashi Yoshida, and Yoshihiko Sako^*^

Graduate School of Agriculture, Kyoto University, Kitashirakawa Oiwake-cho, Sakyo-ku, Kyoto, 606-8502, Japan

*Address correspondence to Yoshihiko Sako: E-mail, sako@kais.kyoto-u.ac.jp; Tel., +81-75-753-6217; Fax, +81-75-753-6226.


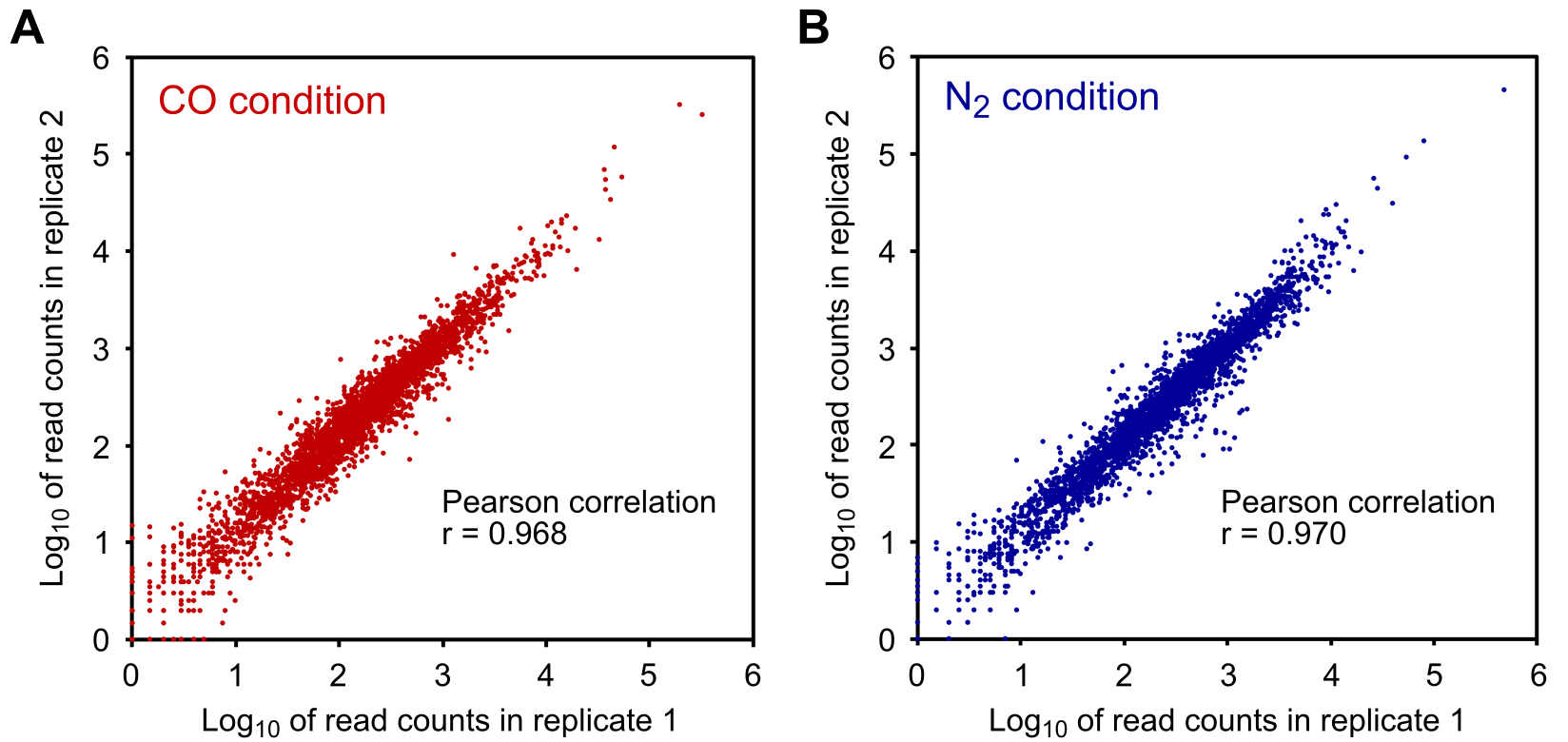


**Fig S1** Comparison of the biological replicates in the RNA sequencing experiments in the presence (A) or absence (B) of carbon monoxide.
